# Molecular Approaches to Improve the Insecticidal Activity of *Bacillus thuringiensis* Cry Toxins

**DOI:** 10.3390/toxins6082393

**Published:** 2014-08-13

**Authors:** Wagner A. Lucena, Patrícia B. Pelegrini, Diogo Martins-de-Sa, Fernando C. A. Fonseca, Jose E. Gomes, Leonardo L. P. de Macedo, Maria Cristina M. da Silva, Raquel S. Oliveira, Maria F. Grossi-de-Sa

**Affiliations:** 1 Embrapa Cotton, Campina Grande, 58428-095, PB, Brazil; E-Mail: wagner.lucena@embrapa.br; 2 Graduate Program in Cellular and Molecular Biology, Federal University of Rio Grande do Sul, Porto Alegre, 91501-970, RS, Brazil; 3 Embrapa Genetic Resources and Biotechnology, Brasília, 70779-917, DF, Brazil; E-Mails: pbpelegrini@gmail.com (P.B.P.); dmartinsdesa@gmail.com (D.M.-S.); fcafonseca@gmail.com (F.C.A.F.); jose_edilson@yahoo.com.br (J.E.G.); leonardo.lima@embrapa.br (L.L.P.M.); cristina.mattar@embrapa.br (M.C.M.S.); raquelsam@gmail.com (R.S.); 4 Department of Molecular Biology, Federal University of Brasília, Brasília, 70910-900, DF, Brazil; 5 Post-Graduation of Genomic Sciences and Biotechnology, Catholic University of Brasilia, Brasília, 70790-160, DF, Brazil

**Keywords:** Cry toxins, insect pests, biocontrol, evolution, phage, display, DNA shuffling, *in silico* studies, specific mutation

## Abstract

*Bacillus thuringiensis* (Bt) is a gram-positive spore-forming soil bacterium that is distributed worldwide. Originally recognized as a pathogen of the silkworm, several strains were found on epizootic events in insect pests. In the 1960s, Bt began to be successfully used to control insect pests in agriculture, particularly because of its specificity, which reflects directly on their lack of cytotoxicity to human health, non-target organisms and the environment. Since the introduction of transgenic plants expressing Bt genes in the mid-1980s, numerous methodologies have been used to search for and improve toxins derived from native Bt strains. These improvements directly influence the increase in productivity and the decreased use of chemical insecticides on Bt-crops. Recently, DNA shuffling and *in silico* evaluations are emerging as promising tools for the development and exploration of mutant Bt toxins with enhanced activity against target insect pests. In this report, we describe natural and *in vitro* evolution of Cry toxins, as well as their relevance in the mechanism of action for insect control. Moreover, the use of DNA shuffling to improve two Bt toxins will be discussed together with *in silico* analyses of the generated mutations to evaluate their potential effect on protein structure and cytotoxicity.

## 1. Introduction

Cry toxins or δ-endotoxins secreted by the gram-positive bacterium *Bacillus thuringiensis* (Bt) are effectively applied to control crop pests and disease vectors due to their specificity and toxicity toward certain insect orders. The mechanism of action of Cry toxins has been extensively reported, and two main theories have been developed to understand how these proteins act on the midgut of several insect species [1,2,3]. The tertiary structures of nine Cry toxins (Cry1Aa, Cry34Ab1, Cry1Ac, Cry2Aa, Cry3Aa, Cry3Bb1, Cry4Aa, Cry5B, and Cry8Ea1) have been determined by X-ray crystallography [4,5,6,7,8,9,10,11], and the structures confirm their conserved organization into three domains. Domain I is composed of seven antiparallel α-helices. The central hydrophobic helix (α5) is surrounded by amphipathic helices [4] and is involved in membrane pore formation. Domain II is organized into three antiparallel β-sheets and two shorter α-helices; this domain is related to receptor binding specificity [12]. Moreover, Domain III includes the *C*-terminus of most of Cry toxins and consists of two antiparallel β-sheets. Domain III, together with domain II, is responsible for structural stability and interaction with insect receptors [6]. Insects exhibit high genetic diversity and acquire resistance to diverse control strategies. Therefore, new approaches for the development of novel molecules with improved insecticidal activity are necessary and represent an important focus for biotechnological strategies. Additionally, the generation of insect resistant transgenic crops is not only one of the most widespread but also the most effective plant biotechnology applications worldwide.

*In vitro* directed evolution to improve the insecticidal activity of Cry toxins has been used with significant success [13,14,15]. In recent years, knowledge of the rate-limiting steps of Cry toxicity in different insect pests has considerably increased. Although the mechanism of action for Cry toxicity has not been completely elucidated, several studies have identified specific binding regions in the Cry toxins. Additionally, several mutagenesis strategies and selection procedures have been described. For example, in DNA shuffling approaches [16,17], the variants of an individual gene or a set of homologous genes are fragmented and recombined to give rise to genes with different base compositions.

Therefore, in this report, we briefly summarize the natural and *in vitro* evolution of Cry toxins and the contributions that improve the insecticidal activity of these toxins at the molecular level. Additionally, we describe the strategies used to obtain Cry variants from two different toxin families: Cry1I and Cry8Ka. Similarly, we discuss preliminary results from *in silico* evaluations of specific mutations from variants obtained using phage display and DNA shuffling techniques on three Cry toxins (Cry1A, Cry1Ia12 and Cry8Ka1) to determine their contribution on improved insecticidal activity and to direct the design of future studies to elucidate the mechanism of Cry toxins.

## 2. Brief Insights into the Natural Evolution of Cry Toxins

Of the Cry toxins studied to date, more than 700 genes have been identified, corresponding to proteins divided into approximately 70 different groups [18,19]. However, phylogenetic analyses have demonstrated that Cry toxins can be divided into four families that do not share phylogenicity [20]. Hence, Cry toxins can be classified as three-Domain crystal toxins (3D-Cry), mosquitocidal Cry toxins (Mtx), binary-like toxins (Bin) and cytolytic (Cyt) toxins [20].

Since the 1990s, the nomenclature of Cry toxins has been based on their amino acid sequence identity, and these toxins are classified according to a three-step procedure [18]. Toxins that share less than 45% identity in their primary sequence are identified by different Arabic numbers in their names. When Cry toxins exhibit amino acid sequence identity between 45% and 70%, a capital letter is included after the number. Moreover, if the sequence identity between Cry toxins is from 70% to 95%, a lowercase letter is added to the toxin name [18]. Cry toxins are also classified according to their specificity toward insect orders and/or nematode species.

Of the four families, 3D-Cry toxins (Cry toxins hereafter) represent the largest number of toxins and comprise over 50 distinct groups [19]. Cry toxins share high amino acid sequence identity and similar tertiary structures. Previous studies on the analysis of phylogenetic trees of protoxins and mature Cry toxins have indicated that, using protoxins for the phylogenetic tree, Cry1 proteins are grouped into one core cluster [21]. Moreover, the branch formed by Cry1 toxins was clearly and sufficiently separate from other protoxins to classify them as a subdivision group in the phylogenetic tree. In contrast, Cry toxin groups, such as the Cry8 and Cry9 protoxins, demonstrated an arrangement that was identical to that of the branch of Cry2 and Cry11 protoxins [21,22]. However, when the phylogenetic tree of active proteins was analyzed, different results were observed. The Cry1B, Cry1Ia and Cry1Ib groups of toxins were not located in the branch of the general Cry1 toxin group but were placed with the cluster of toxins that exhibit insecticidal activity against Lepidoptera insects. This finding was expected, as toxins in the Cry1B, Cry1Ia and Cry1Ib groups exhibit activity against this order of insect pests. In contrast, other Cry1 toxins groups were unexpectedly and phylogenetically near a second cluster of proteins that exhibit insecticidal activity against Coleopteran insects. Therefore, the Cry1I and Cry1B groups may exhibit activity against coleopterans, in addition to the Cry3, Cry7 and Cry8 groups [21,22].

**Figure 1:**
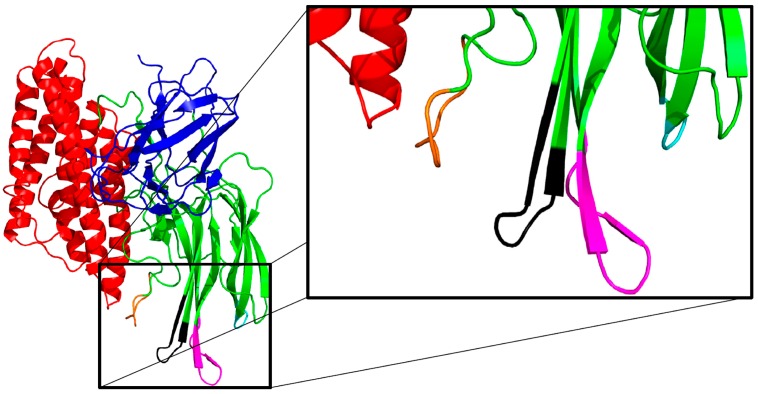
Structure of an activated Cry1Ab toxin. The three Domains are colored as follows: Domain I (red), Domain II (green), and Domain III (blue). Loop 1 is shown in cyan, loop 2 is shown in magenta, loop 3 is shown in black and loop α8 is shown in orange.

When an analysis of each Domain of Cry toxins was performed using phylogenetic trees, interesting results were observed. The tertiary structure of Cry1Ab (Figure 1) reveals three Domains, with Domain I as the most conserved region in Cry toxins. Because this Domain is involved in membrane insertion and pore formation, it is suggested that Cry toxins maintained this region intact despite genetic variability, allowing modifications in other sites of the protein sequence and structure [23,24]. Nevertheless, some mutations that were found in native Bt strains are located in Domain II (specifically at three surface-exposed loops). These loops have been described as important sites for receptor recognition and binding [25,26]. Therefore, the positive selection observed in Loops 1, 2 and 3 may be involved in the increase in specificity and interaction of Cry toxins with the different receptors located at the surface of insect cells [24].

## 3. Mechanism of Action

Active toxins are able to bind receptors at the brush border membrane of midgut insect cells, inducing pore formation and cell death. The toxin/receptor interaction has been extensively characterized [27,28,29,30,31,32,33,34,35,36]. The high amino acid sequence identity among Cry toxins has led to the suggestion of a similar mode of action. However, the efficacy of Cry toxins is dependent on the recognition of a specific receptor, as well as on protein oligomerization and the activation of protoxins by insect proteases [2,25,30]. Different cell surface receptors, such as cadherins, aminopeptidases (APNs) and alkaline phosphatases (ALPs), have been described that recognize Cry toxins and are responsible for the susceptibility of several insect species.

Although extensively studied, the mechanism of action of Cry toxins remains poorly defined, particularly with regard to how specific residues and Domains in these proteins respond following binding to an insect cell receptor. Briefly, it has been suggested that following ingestion by insect pests, Cry toxins are activated after solubilization by midgut proteases and interact with different receptors located at the surface of the epithelial cells [3,30,36,37,38,39,40]. This interaction leads to a conformational change that facilitates protein oligomerization and insertion into the membrane. An increase in membrane permeability follows, resulting in cell death [3,30,36,37,38,39,40].

Another theory suggests that, following binding to a specific cell receptor, Cry toxins stimulate a signaling pathway involving G proteins and adenylyl cyclase, which activate cAMP and protein kinase A, leading to cell and insect death [2].

Previous studies have revealed that the recognition of Cry toxins by cell surface receptors occurs in different regions of the protein. Monomeric active Cry1Ab toxin has been described to interact with ALPs and APNs *via* loop 3 of Domain II and β16 of Domain III. In contrast, the oligomeric form of Cry1Ab binds ALP and APN *via* loop 2 of Domain II [35,41]. In addition, Cry1Ab binds cadherin receptors *via* loops 2 and 3 and α8 of Domain II [42,43]. Therefore, an understanding of the specific regions and residues involved in the mechanism of action of Cry toxins with different cell surface receptors will enable the design of novel molecules with improved activity for insect biocontrol.

## 4. Biotechnology Strategies to Speed *in Vitro* Molecular Evolution of Cry Toxins

Phage display and DNA shuffling techniques have been widely used on Cry toxins to explore specific residues and interaction sites involved in their insecticidal activity. Thus, among Cry1A toxins, phage display assays have been utilized using different phage libraries, such as M13 and T7, although studies using M13 phages have demonstrated greater efficiency than those using T7 phages [44,45,46,47]. Using this approach, novel toxins have been generated that exhibit improved activity toward specific insect pests. Previous studies have reported the construction of a library of Cry1Aa toxin Domain II Loop 2 sequences that was displayed on T7 phages and used to evaluate the binding affinity toward the *Bombyx mori* cadherin receptor [48]. After five rounds of selection, a Cry1Aa mutant containing a mutated Loop 2 in Domain II was obtained that exhibited a 6-fold increase in the insecticidal activity against *B. mori*, indicating that mutations in this region may affect Cry toxin binding to the receptor [48].

Furthermore, Lassner and Bedbrook [49] reported that the spectrum of insects controlled by Bt Cry toxins could be broadened using a DNA shuffling strategy. These authors used Cry1Ca and Cry1Ab, which are parental toxins originally ineffective for the control of *Spodoptera* sp. The resultant Cry1Ca-shuffled library genes were screened for activity against *S. exigua*. A Bt variant improved the activity (3.8 fold) against *S. exigua* in comparison to the original gene sequence, demonstrating the effectiveness of DNA shuffling to improve the insecticidal activity of Cry toxins for the control of insect pests. Using a different strategy, another study demonstrated that shuffling of Cry coding regions is an effective tool to generate diverse chimeric Cry proteins [50]. Moreover, chimeras containing mutations in Domain III of Cry proteins demonstrated higher activity against *Lucilia cuprina* and *Epiphyas postvittana* [50]. A *cry1Ac* gene was modified using error-prone PCR and staggered extension process (StEP) shuffling combined with Red/ET homologous recombination to evaluate the insecticidal activity of Cry1Ac [51]. The Cry1Ac toxin variant screened using an insect bioassay exhibited increased (1.4 fold, LC_50_) insecticidal activity against *S. exigua* larvae, whereas the original insecticidal activity against *Helicoverpa armigera* larvae was retained. Based on these results, these authors concluded that mutations in Domain III of Cry1Ac, particularly those located in the loop between β16 and β17, are essential for the insecticidal activity against insect pests. Using a different method for directed evolution of Cry toxins, consisting of a combination of error-prone PCR, staggered extension process (StEP) shuffling and Red/ET homologous recombination, these authors [51] sought to increase the Cry toxin binding affinity for the insect receptor. Random mutations were introduced in residues in Domain II of the Cry1Aa toxin, the mutant toxins were expressed on phages, and the resultant library was screened using cadherin-like protein-coated beads. Phages expressing abnormal or low-affinity mutant toxins were excluded, and phages with high-affinity mutant toxins were selected. The results indicated that a method combining T7 phage display with selection using cadherin-like protein-coated magnetic beads could be used to increase the activity of Cry toxins [48]. More recently, Fuji *et al*. [52] generated mutant toxins with improved binding affinity against *Bombyx mori* cadherin-like receptor (BtR175) using directed evolution. Four serial residues of Cry1Aa were replaced with random amino acids and were displayed on T7 phages for library construction. Through five cycles of panning of the phage libraries using BtR175, 11 mutant phage clones were selected, and mutant toxin sequences were evaluated. The binding affinities of the three mutants were 42-, 15-, and 13-fold higher than that of the wild type, indicating that mutants with improved binding affinity to cadherin can be easily selected from randomly replaced loop 3 mutant libraries using *in vitro* directed evolution.

Although phage display assays in combination with DNA shuffling have been successfully used to improve Cry toxins, site-directed mutations generated in the variants have also been investigated using *in silico* analyses. To better understand the implications of different mutations on the mechanism of action of Cry toxins, as well as their effect on insect pest control, bioinformatics tools are emerging as the methodology of choice. In time, *in silico* evaluations have transformed from a complementary methodology to an essential technique for the analysis of site-directed mutations, facilitating the selection of the optimal variant for a specific activity against a targeted pest.

## 5. *In Silico* Analyses of Cry and Mutant Toxins for the Control of Economically Important Insect Pests

### 5.1. Cry1A: Effects of Amino Acid Modifications on Receptor Binding and Toxicity

Despite the differences between the models for the Cry toxin mechanism of action, certain common characteristics can be noted. Mutagenesis studies have been used to understand the Cry toxin mechanism of action and as a tool for the discovery of specific sites to increase protein activity against insect pests. The activation of these proteins occurs *via* proteases present in the midgut juice of insect species, as well as by the interaction of Cry toxins with cell surface receptors, such as cadherins, which facilitate the removal of α-helix 1 (α1) of the toxins, allowing their oligomerization and subsequent insecticidal activity [37]. Previous studies have demonstrated that modified Cry1Ab and Cry1Ac, with prior α1 removal, were able to form oligomers *in vitro* in the absence of cadherin receptors, becoming toxic against *Pectinophora gossypiella* [53,54,55]. Additional studies revealed that a V171C mutation in alpha-helix 5 (α5) of Cry1Ab resulted in an increase in toxin activity against *L. dispar* due to a possible enhancement of the Cry1Ab unfolding rate, resulting in easier integration into the membrane [54].

Cadherin receptor-binding sites in Cry toxins have been studied for several insect species. Loops 2 and 3 and α8 from Domain II have been identified among the regions involved in receptor binding [56,57] (Figure 1). Mutagenesis studies have been conducted to understand how these regions interact with the target receptors. A Y445C Cry1Aa mutation reduced binding to the BtR175 receptor, resulting in extremely reduced toxicity to *Bombyx mori* larvae [57], whereas an N372 deletion reduced binding and resulted in low larvae mortality [58]. An N372A mutation in Loop 2 of Cry1Ab exhibited enhanced activity against *Lymantria dispar* in *in vitro* assays. When the mutations A282G and L283S were also introduced, the toxicity against the Lepidoptera insect increased 36 fold in comparison with the wild type protein [58]. However, the loop 2 mutations N372A and N372G did not significantly alter Cry1Ab binding to *Lymantria dispar* brush border membrane vesicles (BBMVs) [58].

The G439D Cry1Ab mutation was found to inhibit binding to *Manduca sexta* BBMVs and increased toxin activity by approximately 150 fold. However, care must be taken while conducting amino acid substitutions because some studies have shown that loops 1, 2, and 3 and α8 in Domain II are extremely flexible and are not dependent on amino acid conservation, which hampers efforts to obtain toxins with increased activity against the insects [52,59]. As another example, loop 3 of Domain II in Cry1Aa was substituted by an identical region in Cry4Aa, which exhibits activity against mosquito species. The variant Cry1 toxin demonstrated identical insecticidal toxicity to *Culex pipiens* larvae [60]. These results indicate that the exchange of loops from Domain II of different Cry toxins can add new specificities to the proteins and serves as a strategic tool for the improvement of toxins against targeted pests.

In addition, mutations in Domain III of Cry toxins have been described. A majority of the studies include the substitution of the entire Domain from one toxin with that of another. When Domain III of Cry1Ab was substituted with Domain III of Cry1C, the variant Cry1Ab toxin exhibited a sixfold enhanced activity against *Spodoptera exigua* compared with Cry1C [61]. Similarly, the exchange of Domain III of Cry1Ia with that of Cry1Ba resulted in a toxin that exhibited a threefold increase in the activity against the coleopteran *Leptinotarsa decemlineata* [62]. Because Cry1Ab is toxic to lepidopteran insects, the introduction of its Domain III into the Cry3Aa toxin provided an efficient *in vitro* activity against *Diabrotica virgifera* (Coleoptera: Chrysomelidae) [63,64]. Therefore, the exchange of Domain III between Cry toxins can serve as an efficient technique to improve the insecticidal activity against several insect pest species. Recently, similar concepts have been applied using more robust strategies, such as phage display and shuffling of *cry* genes*.*

In the pore formation model, the glycosylphosphatidylinositol (GPI)-anchored proteins, APNs and ALPs have been shown to participate in the mechanism of action of Cry1A toxins. The interaction between the monomeric toxin with GPI-anchored proteins occurs via strand β16 in Domain III of ALP [41] and via loop 2 and 3 in Domain II of APN [35,65]. Following oligomerization, binding to GPI-anchored proteins occurs *via* loop 2 in Domain II of the Bt toxin [41]. Site-directed mutagenesis has been used to mutate several residues in Cry1A toxins (Table 1). Girard and coworkers [66] individually replaced 19 residues in helix α4 in Domain I of Cry1Aa. These authors observed that 10 mutants exhibited severely reduced activity against *M. sexta* larvae due to the inability to form pores in the cell membrane. This effect was observed after these residues were substituted by different amino acids [67]. Cry1Ab helices α3, α4, α5 and α6 have been targeted for amino acid substitution. Helix α3 mutants were unable to form oligomers, preventing pore formation on the *M. sexta* larvae cell membrane [68]. Of eight helix α4 mutants, six exhibited a dominant negative phenotype, which competed with the wild-type Cry1Ab toxin for oligomer assembly, resulting in reduced membrane insertion and low mortality of *M. sexta* larvae [69]. In addition, an N135Q Cry1Ac mutation in helix α4 in Domain I resulted in low oligomer assembly, suggesting inhibition of protein pore formation, and low mortality of *M. sexta* larvae [70].

**Table 1:** Site-directed mutations of Cry1Aa, Cry1Ab and Cry1Ac and their influence on toxins’ function against insect pests.

Toxin	Mutation	Region	Characteristic	Molecular effect	Toxicity	Reference
**Cry1Aa** ****	L126C	Helix α4 (domain I)	Reduced capacity to form pores	Low influx of ions	Extremely reduced	[66]
	R127C	Helix α4 (domain I)	Increased capacity to form pores	Increased influx of ions	Slightly reduced	
	M130C	Helix α4 (domain I)	Increased capacity to form pores	Increased influx of ions	Slightly reduced	
	R131C	Helix α4 (domain I)	Highly reduced capacity to form pores	Extremely low influx of ions	Extremely reduced	
	I132C	Helix α4 (domain I)	Highly reduced capacity to form pores	Extremely low influx of ions	Reduced	
	Q133C	Helix α4 (domain I)	Reduced capacity to form pores	Low influx of ions	Extremely reduced	
	F134C	Helix α4 (domain I)	Increased capacity to form pores	Increased influx of ions	Reduced	
	N135C	Helix α4 (domain I)	Highly reduced capacity to form pores	Extremely low influx of ions	Extremely reduced	
	M137C	Helix α4 (domain I)	Increased capacity to form pores	Increased influx of ions	Slightly reduced	
	N138C	Helix α4 (domain I)	Highly reduced capacity to form pores	Extremely low influx of ions	Extremely reduced	
	S139C	Helix α4 (domain I)	Highly reduced capacity to form pores	Extremely low influx of ions	Reduced	
	A140C	Helix α4 (domain I)	Highly reduced capacity to form pores	Extremely low influx of ions	Extremely reduced	
	L141C	Helix α4 (domain I)	Increased capacity to form pores	Increased influx of ions	Slightly reduced	
	T142C	Helix α4 (domain I)	Highly reduced capacity to form pores	Extremely low influx of ions	Extremely reduced	
	A144C	Helix α4 (domain I)	Highly reduced capacity to form pores	Extremely low influx of ions	Extremely reduced	
	I145C	Helix α4 (domain I)	Reduced capacity to form pores	Low influx of ions	Slightly reduced	
	P146C	Helix α4 (domain I)	Reduced capacity to form pores	Low influx of ions	Extremely reduced	
	L147C	Helix α4 (domain I)	Highly reduced capacity to form pores	Extremely low influx of ions	Extremely reduced	
	A149C	Helix α4 (domain I)	Increased capacity to form pores	Increased influx of ions	Slightly reduced	
	R127E	Helix α4 (domain I)	Increased capacity to form pores	Increased influx of ions	Slightly reduced	[67]
	R127N	Helix α4 (domain I)	Increased capacity to form pores	Increased influx of ions	Slightly reduced	
	E128C	Helix α4 (domain I)	Highly reduced capacity to form pores	Extremely low influx of ions	Slightly reduced	
	E129C	Helix α4 (domain I)	Abolished capacity to form pores	n/a	Extremely reduced	
	E129K	Helix α4 (domain I)	Abolished capacity to form pores	n/a	Extremely reduced	
	R131D	Helix α4 (domain I)	Increased capacity to form pores	Low influx of ions	Extremely reduced	
	R131E	Helix α4 (domain I)	Highly reduced capacity to form pores	Low influx of ions	Slightly reduced	
	R131H	Helix α4 (domain I)	Highly reduced capacity to form pores	Low influx of ions	Extremely reduced	
	R131Q	Helix α4 (domain I)	Highly reduced capacity to form pores	Extremely low influx of ions	Reduced	
	D136C	Helix α4 (domain I)	Highly reduced capacity to form pores	Extremely low influx of ions	Extremely reduced	
	D136N	Helix α4 (domain I)	Highly reduced capacity to form pores	Extremely low influx of ions	Extremely reduced	
	D136Y	Helix α4 (domain I)	Abolished capacity to form pores	n/a	Extremely reduced	
	T142D	Helix α4 (domain I)	Abolished capacity to form pores	n/a	Extremely reduced	
	T143D	Helix α4 (domain I)	Abolished capacity to form pores	n/a	Extremely reduced	
	Y445C	Loop 3 (domain II)	Reduced Bt-R175 binding capacity	n/a	Extremely reduced	[57]
**Cry1Ab**	R99E	Helix α3 (domain I)	No oligomer formation	No membrane insertion	Extremely reduced	[68]
	L100E	Helix α3 (domain I)	No significant alteration	No significant alteration	No significant alteration	
	Y107E	Helix α3 (domain I)	No oligomer formation	No membrane insertion	Extremely reduced	
	I200D	Helix α6 (domain I)	No significant alteration	No significant alteration	No significant alteration	
	Y203D	Helix α6 (domain I)	No significant alteration	No significant alteration	No significant alteration	
	R99E	Helix α3 (domain I)	No oligomer formation	No membrane insertion	Reduced	[69]
	E129K	Helix α4 (domain I)	Dominant negative effect	Low capacity to insert into cell membrane	Extremely reduced	
	N135C	Helix α4 (domain I)	Dominant negative effect	Low capacity to insert into cell membrane	Extremely reduced	
	D136N	Helix α4 (domain I)	No competition with wild-type Cry1Ab	Low capacity to insert into cell membrane	Slightly increased	
	A140K	Helix α4 (domain I)	No competition with wild-type Cry1Ab	Low capacity to insert into cell membrane	Slightly increased	
	T142C	Helix α4 (domain I)	Dominant negative effect	Low capacity to insert into cell membrane	Extremely reduced	
	T143D	Helix α4 (domain I)	Dominant negative effect	Low capacity to insert into cell membrane	Extremely reduced	
	D136N, T143D	Helix α4 (domain I)	Dominant negative effect	Low capacity to insert into cell membrane	Extremely reduced	
	E129K, D136N	Helix α4 (domain I)	Dominant negative effect	Low capacity to insert into cell membrane	Extremely reduced	[71]
	G439D	Loop 3 (domain II)	No binding to receptor	Low capacity to insert into cell membrane	No significant alteration	
	V171C	Helix α5 (domain I)	Reduced toxin folding	Increased capacity to insert into cell membrane	Highly increased	
	L157C	Helix α5 (domain I)	Reduced toxin folding	Increased capacity to insert into cell membrane	Increased	
	N372A	Loop 2 (domain II)	Increased biding capacity to BBMVs	n/a	Highly increased	[58]
	N372G	Loop 2 (domain II)	Increased biding capacity to BBMVs	n/a	Highly increased	
	N372del	Loop 2 (domain II)	Highly reduced binding capacity to BBMVs	n/a	Extremely reduced	
	N372A, A282G, L283S	Loop2, Loop α8a, Loop α8 (domain II)	Increased biding capacity to BBMVs	n/a	Highly increased	
	Y153D	Loop between α4 and α5 (domain I)	Weaker membrane insertion	n/a	Reduced	[12]
	G282A, S283L	8 Loop (domain II)	Reduced binding to receptor	n/a	Highly reduced	
	R345A, Y350A, Y351A	Loop 1 (Domain II)	Reduced binding to receptor	n/a	Slightly reduced	
	I373A	Loop 2 (Domain II)	Structure instability	n/a	Reduced	
	F371A, G374A	Loop 2 (Domain II)	Alters binding to membrane	Increase in dissociation from the membrane	Highly reduced	
	F440A, G439A	Loop 3 (Domain II)	Affects binding to receptor	n/a	Highly reduced	
**Cry1Ac**	N135Q	Helix α4 (domain I)	No oligomer formation	No membrane insertion	Extremely reduced	[70]
	Q509A	GalNac binding site (domain III)	Highly reduced binding capacity to APN	n/a	Slightly reduced	[72]
	R511A	GalNac binding site (domain III)	Highly reduced binding capacity to APN	n/a	Slightly reduced	
	Y513A	GalNac binding site (domain III)	Highly reduced binding capacity to APN	n/a	Slightly reduced	
	Q509A	GalNac binding site (domain III)	No significant alteration	n/a	Reduced	[73]
	N510A	GalNac binding site (domain III)	Highly reduced binding capacity to ALP	n/a	Reduced	
	R511A	GalNac binding site (domain III)	No significant alteration	n/a	Reduced	
	Y513A	GalNac binding site (domain III)	Highly reduced binding capacity to ALP	n/a	Extremely reduced	
	W545A	GalNac binding site (domain III)	Highly reduced binding capacity to ALP	n/a	Extremely reduced	
	T524N	Loop β16-β17 (domain III)	n/a	n/a	Increased	[51]

The effect of glycosylation is extremely important for the activity of Cry1Ac toxins [74,75]. It is believed that the binding of loops 2 and 3 is dependent on a rapid and low-affinity interaction between a lectin-like region and *N*-acetylgalactosamine (GalNac) molecules located in APN. The binding of Cry1Ac to *Helicoverpa armigera* has also been shown to be dependent on *N*-linked GalNac molecules [76]. Site-directed mutagenesis was performed on several Cry1Ac residues that form the GalNac binding site or that lie near it. Following alanine substitution, several mutants exhibited reduced binding capacity for APN or ALP, resulting in low *M. sexta* [72] and *H. armigera* larvae [73] mortality rates. The T524N Cry1Ac Domain III mutation resulted in a toxin with improved toxicity to *Spodoptera exigua* larvae that did not significantly differ in its effect against *H. armigera* larvae [51].

### 5.2. Analyses of Cry1Ia12 and Its Variants Generated Using in Vitro Directed Evolution

In recent years, the successful application of recombinant DNA technology using Bt toxins with activity for specific insect pests has been reported, resulting in many insect-resistant varieties from genetic engineering. Different genetically modified (GM) crops, including cotton, maize, potato, tomato, rice, eggplant, soybean and crucifer vegetables, expressing Cry toxins from Bt have been grown worldwide and are an effective tool for pest control.

Studies on insect pests of cotton plants to identify new Cry toxins led to the isolation of a *Cry* gene, whose toxin heterologously expressed in *Escherichia coli* exhibited toxicity against two insect pests, *Anthonomus grandis* and *Spodoptera frugiperda* [77]. This recombinant Cry1Ia12 protein at a concentration of 230 μg/mL resulted in 50% mortality of *A. grandis* larvae, whereas a concentration of only 5 μg/mL resulted in 50% mortality of *S. frugiperda* larvae [77]. In this study, the effect of the recombinant Cry1Ia12 toxin was compared with other Bt toxins, including Cry3Aa, Cry1Aa, Cry1Ac and Cry1Ba. However, none of these Cry toxins exhibited equivalent mortality rates for *A. grandis* as observed for Cry1Ia12 [77].

Although no adverse effects of Cry toxins have been described for humans, safety assessment guidelines have been established to ensure that any product developed from GM crops are safe for human consumption [78,79,80,81]. Furthermore, the development of a GM plant is not only time consuming but requires previous assurances regarding the toxicity and allergenicity of the introduced foreign proteins to ensure its safety for future commercialization. Therefore, *in vivo* assays using Cry1Ia12 have been performed in rats to assess its toxicity against mammals [81]. Studies using Wistar rats fed Cry1Ia12 toxin did not indicate any significant difference in total weight gain, protein digestibility or nitrogen balance in comparison to rats fed a diet without the Bt toxin. Moreover, no differences were observed in serum protein, albumin, urea nitrogen, alanine aminotransferase, aspartate aminotransferase and alkaline phosphatase levels. Even when Cry1Ia12 was administered as a single oral dose at a concentration of 12 mg/animal, no toxicity was observed in the evaluated rats [81]. These results suggest the potential application of Cry1Ia12 toxin for the control of insect pests such as *A. grandis* and *S. frugiperda*.

**Figure 2:**
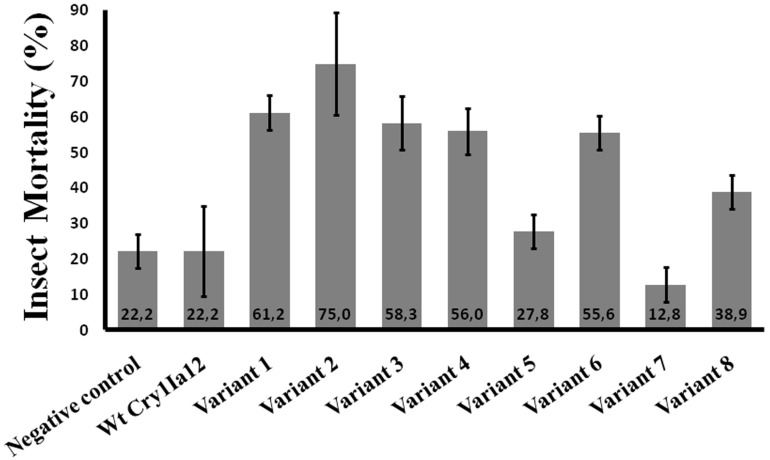
Mortality of *T. l. licus* following ingestion of Cry1Ia12 toxin and its variants (3 µg each). The results represent insect mortality after 5 days of a diet containing each of the respective proteins.

Sugarcane giant borner *Telchin licus licus* (Lepidoptera: Castiniidae) is another important crop pest without an effective management for its control. Therefore, variants of Cry1Ia12 toxin targeting this insect pest have been generated using DNA shuffling and phage display techniques [13]. The Cry1Ia12 toxin exhibits considerable toxicity against the Lepidoptera fall armyworm (*S. frugiperda*) [77], and its potential for toxicity against another Lepidoptera species has been confirmed in a pioneering study in which a DNA shuffling-generated Cry1Ia variant exhibited toxicity to *T. l. licus* [13] (Figure 2). The nucleotide sequence of the original Cry1Ia12 gene was first chemically modified to accommodate plant codon usage. By selecting the clones with increased binding capacity against brush border membrane vesicles (BBMVs), thirty clones were selected for insecticidal activity against *T. l. licus*, of which four resulted in increased mortality of sugarcane giant borer larvae (Figure 2).

**Table 2:** Mutations present in Cry1Ia12 variants. Deletions are indicated by del and insertions by ins.

Variant	Mutation	Domain	Reference
**1**	D233N, E639G	I, III	[13]
**2**	D233N	I	[13]
**3**	I116T, L266F, K580R	I, I, III	[13]
**4**	M45V	*N*-terminus(protoxin)	[13]
	D233N	I	
**5**	S84G, R159K, G380R	I, I, II	This report
**6**	S84G	I	This report
	R159K	I	
	L212del	I	
	S213del	I	
	Q413S	II	
	P414T	II	
	P419L	II	
**7**	S84G	I	This report
**8**	S84G	I	This report
	R159K	I	
	G380R	II	
	K427ins	II	

**Figure 3:**
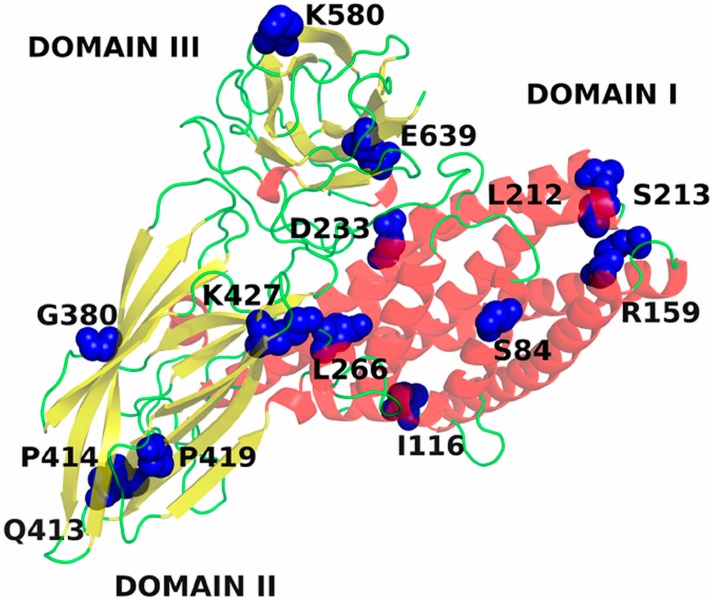
Tertiary structure of Cry1Ia12. α-Helices colored red correspond to Domain I of the toxin. β-Sheets colored yellow color correspond to Domains II and III. Blue spheres represent the mutated residues from DNA shuffling involved in binding to receptors.

**Figure 4:**
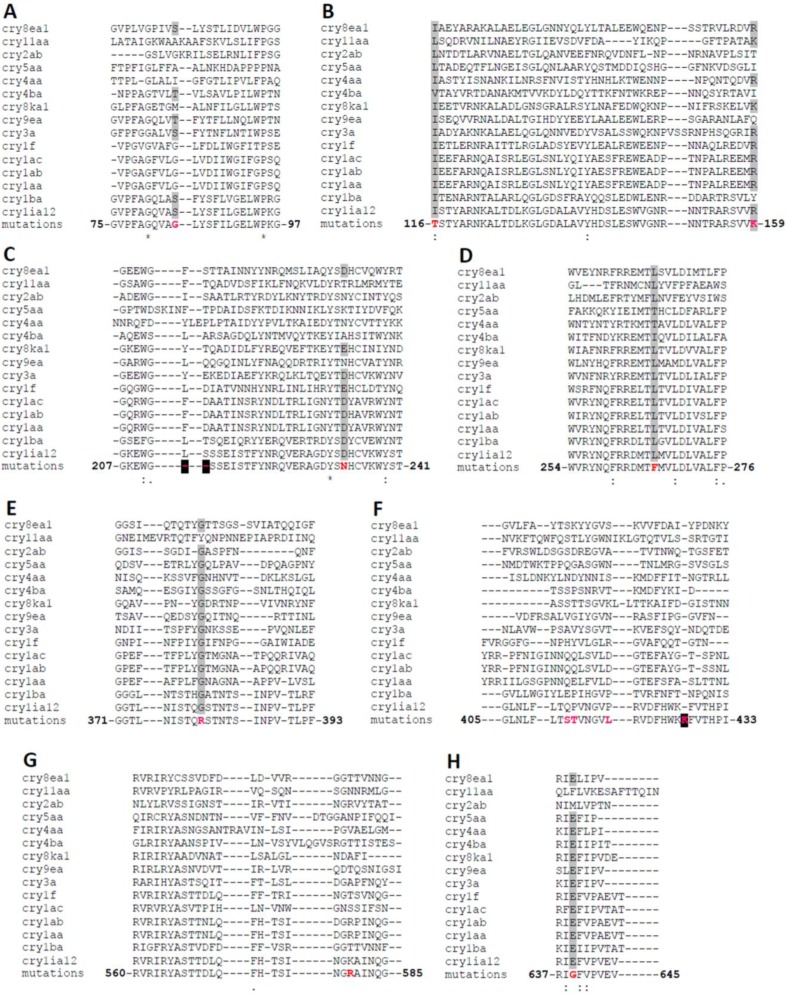
Alignment of 15 Cry toxins: Cry1Aa (AAP40639.1), Cry1Ab (AEV45790.1), Cry1Ac (ACC86135.1), Cry1Ia12 (ADB02877.1), Cry1F (ACD50893.1), Cry1Ba (AAK63251.1), Cry11Aa (YP_001573776.1), Cry8Ea1 (AAQ73470.1), Cry8Ka1 (ACQ99188.1), Cry3A (ABY49136.1), Cry9Ea (ADE60738.1), Cry2Ab (ACC86136.1), Cry4Aa (YP_001573833.1), Cry4Ba (YP_001573790.1) and Cry5Aa (Q45760.1). Polarity conserved residues in mutation regions are highlighted in gray, and residues mutated by DNA shuffling in Cry1Ia12 are depicted in bold red [13] and bold magenta (unpublished results). Deletions or additions of residues are further highlighted in black. Inserts **A**, **B** **C**, **D**, **E**, **F**, **G** and **H** correspond to different regions in the alignment.

**Figure 5:**
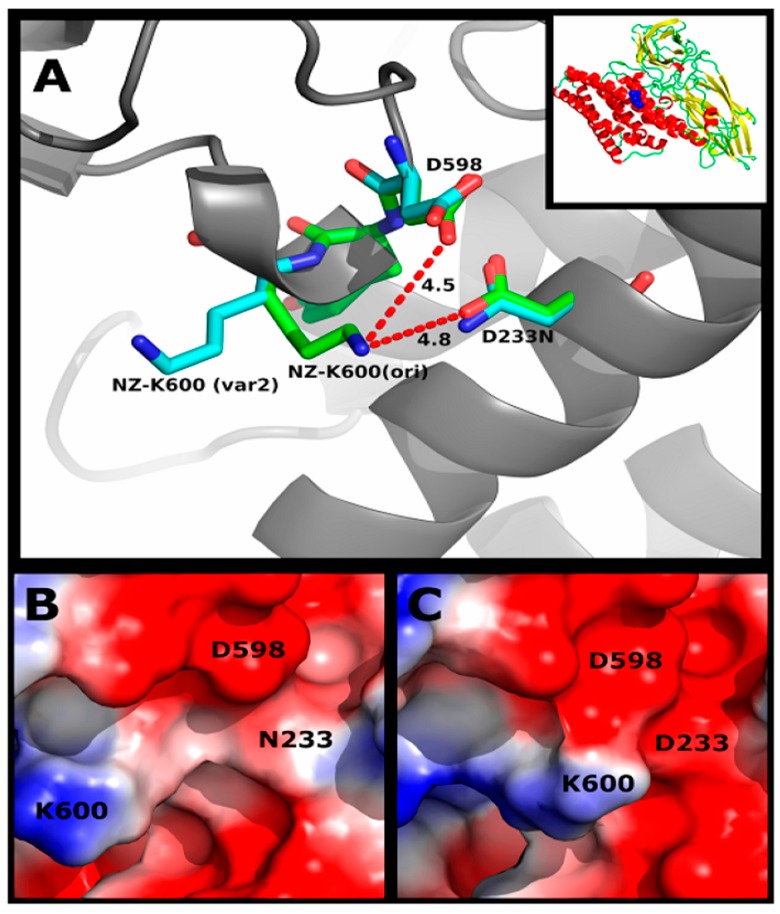
Detailed view of D233 from variant 2. (**A**) Distance between the NZ atom of K600 and OD2 of D233 (4.8 Å) in wild-type Cry1Ia12; (**B**) ABPS-SEP of wild-type Cry1Ia12 depicting the proximity between K600, D598 and D233; (**C**) ABPS-SEP of variant 2 depicting the displacement of K600 from the salt bridge interaction with N233 and D598. The inset in the upper right corner represents the entire Cry1Ia12 structure.

Additional analysis identified six different mutations present in the selected Cry1Ia12 variants: D233N, E639G, I116T, L266F, K580R and M45V (Table 2) [13]. However, the D233N mutation (Figure 3 and Figure 4) is present in three of the variants, and the authors concluded that this mutation was most likely responsible for 75% of the *T. l. licus* mortality level in bioassays*,* as shown for variant 2 (Figure 2 and Figure 5) [13].

To better understand the role of each amino acid substitution in the toxin structure and the consequences for receptor interactions, the amino acid sequences of 15 Cry toxins were aligned, six of which are classified as belonging to the Cry1 family. The structural alignment indicated that all amino acid substitutions, with the exception of K580R, are conserved in all Cry1 toxins analyzed (Figure 4). Therefore, these regions may play an important role in stabilization of the protein structure, oligomerization, membrane insertion, receptor recognition or binding. A majority of the substitutions occurred in Domain I, which has been related to oligomerization and membrane insertion [82]. Dean and coworkers (1996) introduced amino acid substitutions into Cry1Ab toxins (Y153D, Y153A and Y153R) using site-directed mutagenesis. These authors concluded that the negatively charged aspartate is less favorable for membrane insertion in Cry1A family toxins than positively charge residues, such as arginine, and non-polar residues, such as alanine. This report may also be applied to the D233N mutation identified in variant 2 (Cry1Ia12 toxin), as this protein contains only this mutation in its sequence and exhibited the highest increase in cytotoxicity among the selected variants. Hence, the structural implications of substituting an oxygen atom for a nitrogen atom in the tertiary toxin structure have been investigated. For D233 in wild-type Cry1Ia12 and N233 in variant 2, the respective side chains were found to be solvent exposed, and these residues lie close to β-strand 20 in Domain III [13]. The local charge difference caused by nitrogen substitution in variant 2 affected the interaction of D233 with the residues D598, Y599 and K600. Interestingly, the D233N mutation promoted the displacement of K600, which is solvent exposed in variant 2 (Figure 5). Whether K600 in the loop between β19 and β20 is involved in receptor recognition or membrane insertion remains unclear. D233 is conserved in Cry1 toxins (Figure 5C), and the influence of asparagine substitution on Bt toxin binding to other Lepidoptera cadherin-like receptors is currently unknown. To address this question, studies on the corresponding residue in Cry1Ab, D205, have been performed using *in silico* interaction models. Although preliminary, these results demonstrate the importance of D233 in the Cry1Ia12 mechanism of action and strongly indicate that this region is sensitive to point mutations, playing an important role in cytotoxicity. In addition, because D233 is located in α-helix 6 in Domain I, it is known to be essential for toxin cell membrane insertion following receptor recognition and oligomerization [36]. If our conclusions are correct, it is likely that this mutation of a highly conserved negatively charged amino acid can improve or induce toxicity in other Cry1 toxins, which have previously been shown to specifically bind BBMVs of target insects. Investigation of this approach in other insect orders may be valuable.

**Figure 6:**
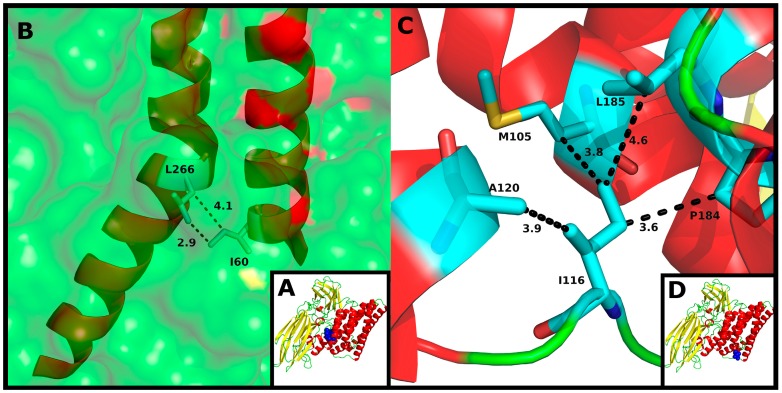
(**A**) Tertiary structure of Cry1Ia12. Domain I is colored red, whereas Domains II and III are colored yellow; (**B**) Detailed view of the interaction between L266 within the toxin and I60 in helix α-1. Distances are shown as dashed lines and are measured in angstroms (Å). α-Helix secondary structure is represented in red, and the protein surface is represented in green; (**C**) Detailed view of I116 interactions with the hydrophobic residues M105, A120, P184 and L185 in Domain I. Distances are shown as dashed lines and measured in angstroms (Å). α-Helices are colored red, and loops are colored green; (**D**) Tertiary structure of Cry1Ia12. Domain I is colored red, whereas Domains II and III are colored yellow.

Variant 3 of Cry1Ia12 has also been investigated. Of the variants, only variant 3 lacks the D233N mutation and contains three unique substitutions: I116T, L266F and K580R [13]. The residue I116 is located in a solvent-exposed loop in Domain I of Cry1Ia12, and its neutral charge is well conserved among all 15 Cry toxins analyzed (Figure 4B). Although it is inserted in a solvent vulnerable region, I116 remains primarily oriented toward other hydrophobic residues in the toxin, such as M105, A120, P184 and L185 (Figure 6C,D). This observation suggests that mutation to a polar residue, such as threonine, may disturb the environment, exposing the residue toward the solvent and allowing additional intermolecular interactions. Due to the lack of accurate information regarding the mechanism by which Cry toxins form pores and insert in the membrane, the authors refrained from further discussion on the implications of this mutation on Cry1Ia12 cytotoxicity against *T. l. licus.* However, indirectly, Cry1Ab A92 residue, which corresponds to A120 in Cry1Ia12, has been reported to be involved in membrane insertion [83] and is located in α-helix 3, which has recently been reported to be directly involved in oligomer formation [84].

Regarding L266 (Figure 4D and Figure 6B), this residue is buried inside the toxin and interacts, among other hydrophobic residues, with the N-terminal I60 residue in α-helix 1 (Figure 6A,B). Cleavage of α-helix 1 has been shown to induce oligomerization [56,85]. Although the L266F mutation may favor interaction with other hydrophobic residues, the aromatic side chain of phenylalanine is sufficiently large to sterically perturb the environment and displaces the surrounding residues. This feature may lead to a higher degree of freedom for α-helix 1 and, consequently, facilitate its cleavage.

In addition, the K580R mutation (Figure 4G) is located in Domain III. This Domain has been associated with protection from proteolysis, receptor recognition and pore formation [86]. This residue is exposed and interacts with N578 (Cry1Ia12) in the loop between β18 and β19. In Cry1Ac, N578 corresponds to N546 and has been reported to be important for BBMV binding in *H. armigera* [87]. Because both lysine and arginine are positively charged, any influence on receptor binding or cytotoxicity must be attributed to the larger size of the arginine side chain.

Furthermore, other Cry1Ia12 variants have been generated using DNA shuffling and phage display, and the mutations were subsequently analyzed. Considering the variants that specifically interacted with *T. l. licus* BBMVs (data not shown), only one variant (variant 6, Table 2) resulted in an increase in the mortality rate during bioassays against *T. l. licus* larvae (Figure 2). Interestingly, this variant was the only one containing mutations in Loop 2 of Domain II (Q413S, P414T and P419L in Figure 4F), which has been related to receptor binding (this report) and has been extensively investigated. Nevertheless, Loop 2 apparently plays a key role in cytotoxicity [59] rather than binding. Hence, these observations support the importance of Loop 2 in cytotoxicity based on the interaction of this variant with *T. l. licus* BBMVs that was found to be similar to the interaction observed for wild-type Cry1Ia12 (data not shown). All other substitutions caused similar toxicity results compared to wild-type Cry1Ia12 and were excluded from additional analysis (Figure 2). Overall, these evaluations suggest that DNA shuffling contributes to the development of novel Bt toxins with higher toxicity against targeted insect pests. Moreover, the analyses indicated the participation of new regions that can help in the clarification of the mechanisms by which Bt toxins bind, oligomerize and insert into cell membranes to assess the validity of previously described functional models. Therefore, DNA shuffling strategies for the generation of novel molecules can be applied in the development of plant resistance against insect pests for biotechnological control, including resistance against the sugarcane giant borer.

### 5.3. Use of Cry8 Toxins as a Strategic Tool against Coleopteran Insect Pests

The utilization of shuffling and phage display techniques has also been applied to a Cry8Ka1 toxin, which exhibited activity against the cotton boll weevil in *in vitro* assays [88]. Previously, the *cry8ka1* gene was isolated from a collection of *B. thuringiensis* of the Embrapa Genetic Resources and Biotechnology (Brasilia, Brazil) and used to generate novel mutated Bt toxins using DNA shuffling and phage display, as previously described*.* Hence, of 10^5^ variants produced after the fifth round of biopanning, thirty screened variants showed expression signals on dot blot assays and were selected for toxicity bioassays against *A. grandis* [14]. The variants Cry8Ka3 and Cry8Ka5 demonstrated a significant ability to reduce the survival of *A. grandis* larvae at concentrations of 2.83 μg/mL and 8.93 μg/mL, respectively [14]. Analyses of the changes in the amino acid sequence indicated that modifications were distributed along all the Domains of the Cry8Ka5 protein compared with Cry8Ka1. One mutation was observed in Domain I, two mutations in Domain II and three mutations in Domain III of Cry8Ka5. Furthermore, one deletion was detected at the *N*-terminus of the protein, which resulted in a decrease in the size of Cry8Ka5 in comparison to Cry8Ka1. Although these modifications were observed in the variant protein, no changes were observed in the final structure. However, the mutations, particularly R271S, were essential for its toxicity against *A. grandis* [14].

Initial attempts to identify interacting receptors at the cell surface of insect pests were subsequently performed for Cry8Ka5. Ligand blot experiments were performed using one- and two-dimensional gel electrophoresis. Following *de novo* sequencing, two interacting proteins were identified: a heat-shock cognate protein (HSP) and a vacuolar ATPase (V-ATPase). These proteins have not been previously described as major Bt toxin interaction partners, in contrast to APNs and cadherins, and appear to play indirect roles in the toxicity of Cry8Ka5 within the *A. grandis* midgut [88].

Recently, additional *in silico* analyses have been performed on the Cry8Ka5 structure to better understand its mechanism of action against insect pests. Hence, in comparison to the original toxin Cry8Ka1, the mutations in Cry8Ka5 are not located within conserved motifs between both proteins. Because these motifs are related to the structural stability of Cry toxins, it is suggested that both toxins demonstrate an identical global conformational behavior [18,34,89].

Furthermore, a deletion of 16 residues at the *N*-terminus of Cry8Ka5 has been identified [35]. Nevertheless, this reduction in protein size did not affect Cry8Ka5 activity, as this deletion lies outside of the activation site of the Cry8Ka1 protoxin.

The mutation of an arginine to an asparagine residue at position 82 has been found in the middle portion of α-helix 3 (Domain I) and near other residues involved in oligomerization and/or membrane insertion [66,67,71,82,90,91,92,93]. In Cry1A toxins, some arginine residues in α-helix 3 participate in intramolecular salt bridges that contribute to the structural stability of Domain I [92]. However, in Cry8Ka1, the side chain of the solvent-exposed R82 lies in a position away from other potential salt bridge partners (aspartate and glutamate residues). This finding suggests that this residue interacts more strongly with the solvent. Cry8Ka5, whose toxicity is threefold higher than Cry8Ka1, contains a glutamine residue at position 82, and the loss of a positively charged residue may interfere with membrane interaction or with oligomerization.

Moreover, other aromatic residues can commonly substitute tyrosine residues without compromising protein function [94]. In the case on Cry8ka5, the substitution Y260C is located in a long loop composed of 55 residues, which also contains α-helix 8 and another smaller α-helix. This variable region of Domain II is directly involved in Cry toxin specificity, as this region binds insect cadherin-like receptors [34,95,96]. Furthermore, this loop exhibits high flexibility, and α-helix 8 exhibits the propensity to lose its secondary structure, as observed from molecular dynamics simulations. Although the substitution of Y260 with a cysteine residue did not result in an effective modification of the local electrostatic surface potential, the structural changes arising from different side chain topologies may influence intermolecular interactions, particularly those involving Ca^2+^ present in cadherins.

Generally, arginine and glycine can be involved in binding phosphate groups [97,98]. However, the glycine side chain confers higher flexibility to loops in comparison to arginine. The R508G mutation is located in a polymorphic region near β-sheet 18, which is the fourth conserved region among Cry toxins [89]. This region is rich in arginine and tyrosine residues and has been reported to interact with GPI-APN and GPI-ALP in the presence (as in Cry1Ac) or absence of Gal-NAc (as in the Cry toxins studied in this report) [34,99]. Therefore, mutations in this loop may likely affect interactions with receptors and result in an increase in flexibility due to the presence of a glycine residue flanking β-sheet 18 at position 508. This feature can also facilitate the binding of the modified toxin (Cry8Ka5) with *A. grandis* membrane receptors*.*

**Figure 7:**
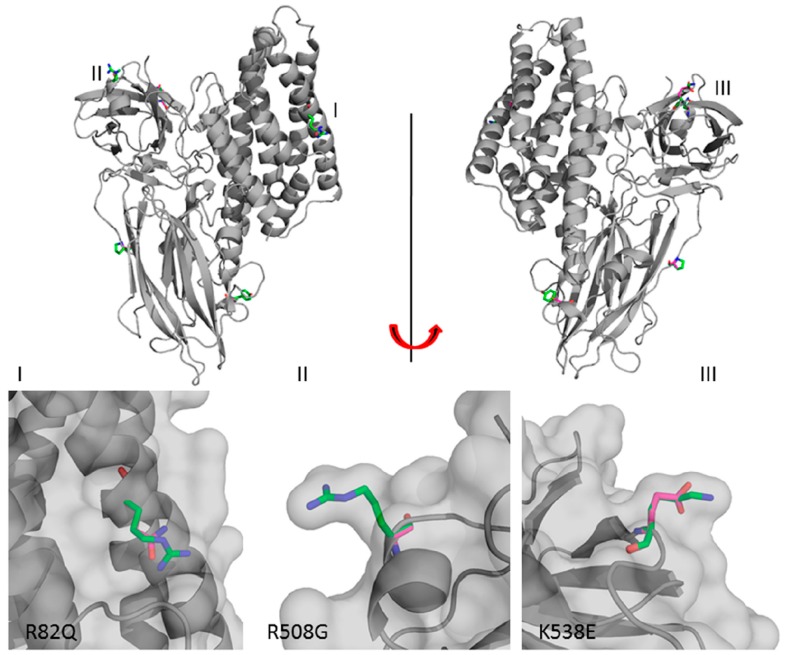
The tertiary structure of Cry8Ka5. Roman numbers indicate the Domains of the Cry toxin. Mutated residues (R82Q, R508G, and K538E) observed in the three Domains are shown in a detailed view.

The K538E mutation is located in a loop situated between β-sheets 20 and 21 of Domain III, which is exposed toward the solvent. This mutation promotes a substantial modification with the exchange of a positively charged residue with a negatively charged residue. In addition, in Cry8Ka1, K538 participates in a salt bridge with D520, which is located at the extreme loop of β-sheet 19. However, in Cry8Ka5, the negative charge of glutamate (E538) in this region disrupts the interaction with D520 (Figure 7). In the loops of the three Domains, some residues have been characterized as important for Cry protein toxicity due to their involvement in the interaction with insect receptors [72,87,99,100]. Domain III is the smallest of the three Domains and has been reported to function as a receptor binding site for GPI-ALP, GPI-APN [41,65] and Gal-NAc [31]. Therefore, the K538E mutation may modify the affinity for GPI-anchored proteins from *A. grandis* and may contribute to the enhanced toxicity of Cry8Ka5, which has been previously inferred from bioassays.

Although precise structural characterization of the interactions between Cry toxins and Coleopteran membrane receptors is lacking, using the details reported for Lepidoptera and Diptera receptors, it is possible to identify the regions of Cry8Ka5 that confer toxicity from previous results reported for the Cry1A and Cry3Aa toxins. From these details, it is possible to estimate the contribution of the previously described mutations to insect cell toxicity.

## 6. Perspectives of Biotechnological Application of Novel Molecules Selected by *in Vitro* Directed Evolution for Insect Pest Control

Currently, over 60 commercially approved for market worldwide transgenic events have been developed to provide resistance against several insect pest species. Of these transgenic events, 41 contain toxins from the Cry1A family (Table 3). In contrast, events containing proteins from the Cry1I or Cry8 families have not yet been reported [101]. Lepidoptera insects are the primary target for all transgenic plants to date, with only six events exhibiting resistance against Coleopteran pests, which are restricted to the *Diabrotica* species and have been exclusively developed for maize plants. The first transgenic plants released to the market have already lost their effectiveness due to the development of insect resistance to Cry toxins present in transformed plants over the last 20 years. Therefore, new toxins have been inserted into plant species to restore their ability to control pest growth. Hence, events with two or more Cry toxins serve different purposes: (i) to decrease the rate of insect development of evolutionary features that inhibit Cry toxin activity for pest control (plant pyramid) and (ii) to increase plant resistance against different insect species at once.

**Table 3:** Transgenic plants expressing toxins from Cry1A family for insect resistance. Source: [102].

Plant	Company	Event	Year/Country regulatory approval	Toxin(s) inserted	Insect species for resistance
**Soybean**	Monsanto Company	MON87701	Canada (2010); United States (2011)	Cry1Ac	*Anticarsia gemmatalis*, *Pseudoplusia includens*
		MON87701/MON89788	Colombia (2012)	Cry1Ac, CP4 epsps	Lepidopteran pests
**Cotton**	Dow AgroSciences LLC	3006-210-23	Mexico (2004); United States (2004); Canada, Japan (2005)	Cry1Ac	*Heliothis virescens*, *Helicoverpa zea*, *Pectinophora gossypiella*, *Spodoptera exigua*
	Calgene Inc.	31807/31808	United States (1997/1998); Canada, Japan (1998/1999)	Cry1Ac	Lepidopteran pests
	Syngenta Seeds, Inc.	COT67B	Australia, United States (2009)	Cry1Ab	*Helicoverpa zea*, *Heliothis virescens*
	Dow AgroSciences LLC	DAS-21Ø23-5/DAS-24236-5	Mexico, United States (2004); Australia (2005); Japan (2005); Korea (2005/2008); Brazil (2009)	Cry1Ac, Cry1F	*Helicoverpa zea*, *Heliothis virescens*, *Pectinophora gossypiella*
		DAS-21Ø23-5/DAS-24236-5/MON-Ø1445-2	Mexico (2005); Korea (2006); Japan (2006)	Cry1Ac, Cry1F	*Helicoverpa zea*, *Heliothis virescens*, *Pectinophora gossypiella*
		DAS-21Ø23-5/DAS-24236-5/MON88913	Japan, Korea, Mexico (2006)	Cry1Ac, Cry1F	*Helicoverpa zea*, *Heliothis virescens*, *Pectinophora gossypiella*
	JK Agri Genetics LTd (India)	Event-1	India (2006)	Cry1Ac	*Earias vittella*, *Helicoverpa armigera*, *Pectinophora gossypiella*
	Bayer CropScience	LLCotton25/MON15985	Japan (2006/2007); Korea (2007/2008); Mexico (2008)	Cry1Ac, Cry2Ab	*Helicoverpa zea*, *Heliothis virescens*, *Pectinophora gossypiella*
	Monsanto Company	MON15985	Australia, United States (2002); Japan (2002/2003); Canada, Mexico, Philippines, South Africa (2003); Korea (2003/2004) European Union (2005); China, India (2006); Burkina Faso (2008); Brazil, Colombia (2009)	Cry1Ac, Cry2Ab2	*Helicoverpa zea*, *Heliothis virescens*, *Pectinophora gossypiella*
		MON-15985-7/MON-Ø1445-2	Australia (2002); Korea (2004/2008); Philippines (2004); European Union, Japan (2005); Mexico (2006)	Cry1Ac, Cry2Ab	*Helicoverpa zea*, *Heliothis virescens*, *Pectinophora gossypiella*
		MON-ØØ531-6/MON-Ø1445-2	Mexico (2002); Australia (2003); Japan, Philippines (2004); Korea (2004/2008); European Union, South Africa (2005); Colombia (2008); Argentina, Brazil (2009)	Cry1Ac	*Helicoverpa zea*, *Heliothis virescens*, *Pectinophora gossypiella*
**Cotton**	Monsanto Company	MON15985/MON88913	Japan (2005/2006); Australia, Mexico, Philippines (2006); Korea (2006/2008); South Africa (2007); Colombia (2010)	Cry1Ac, Cry2Ab	*Helicoverpa zea*, *Heliothis virescens*, *Pectinophora gossypiella*
		MON531/757/1076	United States (1995); Australia, Canada (1996); Japan, Mexico, South Africa (1997); Argentina (1998); India (2002); Colombia (2003); Korea (2003/2004); China, Philippines (2004); Brazil, European Union (2005)	Cry1Ac	*Helicoverpa zea*, *Heliothis virescens*, *Pectinophora gossypiella*
**Tomato**	Monsanto Company	5345	United States (1998); Canada (2000)	Cry1Ac	*Helicoverpa zea*, *Heliothis virescens*, *Pectinophora gossypiella*
**Maize**	Syngenta Seeds, Inc.	176	United States (1995); Canada (1995/1996); Japan (1996); Argentina (1996/1998); European Union, The Netherlands, Switzerland, United Kingdom (1997); Australia, South Africa (2001); Philippines (2003); Korea (2003/2006); China, Taiwan (2004)	Cry1Ab	*Ostrinia nubilalis*
		BT11 (X4334CBR, X4734CBR)	Canada, Japan, United States (1996); European Union, Switzerland, United Kingdom (1998); Argentina, Australia (2001); South Africa (2002); Korea (2003/2006); Russia (2003); China, Taiwan, Uruguay (2004); Philippines (2005); Brazil, Mexico (2007); Colombia (2008/2009)	Cry1Ab	*Ostrinia nubilalis*
		BT11/GA21	Canada (2005); Korea (2006/2008); Japan, Mexico, Philippines (2007); Argentina, Brazil (2009); Uruguay (2011); Colombia (2012)	Cry1Ab, Vip3Aa20	*Agrotis ipsilon*, *Ostrinia nubilalis*, *Helicoverpa zea*, *Spodoptera frugiperda*
		BTT11/GA21/MIR162	Brazil (2011); Colombia (2012)	Cry1Ab, Vip3Aa20	*Helicoverpa zea*, *Spodoptera frugiperda*, *Agrotis ipsilon*
		BT11/MR162	United States (2009)	Cry1Ab, Vip3Aa20	*Agrotis ipsilon, Ostrinia nubilalis*, *Helicoverpa zea*, *Spodoptera frugiperda*, *Spodoptera albicosta*
**Maize**	Syngenta Seeds, Inc.	BT11/MIR162/MIR604	United States (2009)	Cry1Ab, Vip3Aa20	*Ostrinia nubilalis*, *Diatraea crambidoides*, *Spodoptera frugiperda*, *Pseudaletia unipunctata*, *Spodoptera exigua*, *Agrotis ipsilon*, *Striacosta albicosta, Diatraea saccharalis*, *Diabrotica virgiferaDiabrotica barberi*, *Papaipema nebris*
		BT11/MIR162/MIR604/GA21	Colombia (2012)	Cry1Ab, mCry3A,Vip3a20	*Diabrotica* spp., *Helicoverpa zea*, *Ostrinia nubilalis*, *Spodoptera frugiperda*, *Agrotis ipsilon*
		BT11/MIR604	Canada, Japan, Korea, Mexico, Philippines (2007); Colombia (2012)	Cry1Ab, mCry3A	*Diabrotica* spp., *Ostrinia nubilalis*
		BT11/MIR604/GA21	Canada, Japan (2007); Korea, Mexico, Philippines (2008);	Cry1Ab	*Diabrotica* spp, *Ostrinia nubilalis*
	DeKalb Genetics Corporation	DBT418	Canada, United States (1997); Japan (1999); Australia (2002); Philippines, Taiwan (2003); Korea (2004)	Cry1Ac	*Ostrinia nubilalis*
	Monsanto Company	GA21/MON810	Japan, South Africa (2003); Korea, Philippines (2004); European Union (2005)	Cry1Ab	*Ostrinia nubilalis*, Other Lepidoteran pests
		MON80100	United States (1996)	Cry1Ab	*Ostrinia nubilalis*
		MON802	United States (1996/1997); Canada, Japan (1997)	Cry1Ab	*Ostrinia nubilalis*
		MON809	Canada, United States (1996); Japan (1997/1998)	Cry1Ab	*Ostrinia nubilalis*
		MON810	United States (1995/1996); Japan (1996/1997); Canada, South Africa (1997); Argentina, European Union (1998); Australia, Switzerland (2000); Mexico, Philippines, Taiwan (2002); Korea (2002/2004); Colombia, Uruguay (2003); China (2004); Brazil (2007)	Cry1Ab	*Ostrinia nubilalis*
		MON810/LY038	Philippines (2006); Japan (2007)	Cry1Ab	*Ostrinia nubilalis*
		MON810/MON88017	Japan (2005); Canada, Korea, Mexico (2006); Taiwan (2009); Colombia (2011)	Cry1Ab, Cry3Bb1	*Ostrinia nubilalis*, *Diabrotica virgifera*
**Maize**	Monsanto Company	MON863/MON810	Japan, Korea, Philippines (2004); European Union (2005); Mexico (2006)	Cry1Ab, Cry3Bb1	*Ostrinia nubilalis*, *Diabrotica* sp.
		MON863/MON810/NK603	Canada, Japan, Korea (2004); Philippines (2004/2005); Mexico (2006); Taiwan (2009)	Cry1Ab, Cry3Bb1	*Ostrinia nubilalis*, *Diabrotica virgifera*
		MON89034	Japan, United States (2007/2008); Australia, Canada, Taiwan (2008); Brazil, European Union, Korea, Philippines (2009); Colombia (2010)	Cry1A.105,Cry2Ab	*Ostrinia* sp., *Diabrotica* sp.
		MON89034/MON88017	Japan (2008); Korea, Philippines, Taiwan (2009); Argentina (2010); Colombia (2011)	Cry1A.105,Cry2Ab, Cry3Bb1	*Diabrotica virgifera,* Lepidopteran pests
	Monsanto Company and Mycogen Seeds c/o Dow LLC	MON89034/TC1507/MON88017/DAS-59122-7	Canada, Japan, Korea, Taiwan, United States (2009); Colombia, Mexico, Philippines (2010)	Cry1A.105, Cry1Fa2, Cry2Ab, Cry3Bb1,Cry34Ab1, Cry35Ab1	*Ostrinia nubilalis*, *Helicoverpa zea*, *Spodoptera frugiperda*, *Agrotis ipsilon*
	Monsanto Company	NK603/MON810	Canada (2001); Japan, Korea, Mexico, Philippines (2004); Argentina (2005/2007); European Union (2007); Taiwan, Brazil, Colombia, El Salvador (2009); Uruguay (2011)	Cry1Ab	*Ostrinia nubilalis*, Lepidopteran pests
	Bayer CropScience (Aventis CropScience (AgrEvo))	T25/MON810	Japan (2003); Colombia (2012)	Cry1Ab	*Ostrinia nubilalis*, Lepidopteran pests
	DuPont Pioneer	TC1507/MON810	Brazil (2011); Colombia (2012); Argentina (2013)	Cry1Ab,Cry1Fa2	Lepidopteran pests
		TC1507/MON810/NK603	Canada (2011); Colombia (2012); Argentina (2013)	Cry1Ab, Cry1Fa2	Lepidopteran pests

Nevertheless, one successful approach for plant resistance against targeted pests, as well as to retard insect resistance toward Cry toxins, is the use of proteins with specific mutated sites. Whether these mutations are selected from DNA shuffling or *in silico* analyses, Cry toxin mutants are innovative and efficient tools that can be applied to transformed plants for insect control.

The purpose of using molecular evolution techniques, such as DNA shuffling and *in silico* analyses is to copy the natural design procedures and accelerate them using *in vitro* direct selection methodologies for a single goal. It is already known that complex linear sequences can rapidly pass through evolution by recombination between individuals, whether it includes high or low levels of point mutagenesis [16,102]. Thus, the use of improved phage display selection of DNA-shuffled libraries, together with *in silico* analyses, has been innovative, and these techniques represent potentially effective tools for the development of new molecules with increased activity against insect pests. The application of mutant Cry toxins for plant transformation and acquisition of different cultivars expressing these foreign proteins present three major advantages (in comparison to the use of insecticide molecules extracted from natural resources): (i) the ability to use only one molecule with enhanced activity against insect pests or a molecule that shares features from two different molecules; (ii) knowledge of the specific location of mutations present in the novel protein facilitates the study and verification of its effects against insect pest receptors, as well as its mechanism of action in the targeted cells; and (iii) the exposure of a mutated molecule can retard the development of resistance and coevolution by insect pests.

Therefore, proteins from other Cry families, such as Cry1I and Cry8, are potential molecules for the development of plant species resistant to other insect pests, such as Coleopterans and the sugarcane giant borer. In conclusion, the use of combined DNA shuffling and *in silico* evaluations can accelerate the design of point mutations, which will allow directed evolution of targeted molecules for biocontrol in agribusiness. Beyond biotechnological applications, these techniques will contribute to a decrease in the use of chemical pesticides in agriculture, consequently leading to a significant enhancement in crop production, as well as facilitate an understanding of the mechanism of action of Cry toxins, which may reveal new insights into the technological development of pest control.

## Abbreviations

LC_50_: the concentration of toxins used to present 50% of its total insecticide activity.
